# Preservation of Partially Mixed Selectivity in Human Posterior Parietal Cortex across Changes in Task Context

**DOI:** 10.1523/ENEURO.0222-19.2019

**Published:** 2020-03-04

**Authors:** Carey Y. Zhang, Tyson Aflalo, Boris Revechkis, Emily Rosario, Debra Ouellette, Nader Pouratian, Richard A. Andersen

**Affiliations:** 1 Department of Biology and Biological Engineering, California Institute of Technology, Pasadena, California 91125; 2 Tianqiao and Chrissy Chen Brain-Machine Interface Center, Chen Institute for Neuroscience, California Institute of Technology, Pasadena, California 91125; 3 Casa Colinas Hospital and Centers for Healthcare, Pomona, California 91767; 4 Department of Neurological Surgery, Los Angeles Medical Center, University of California, Los Angeles, Los Angeles, California 90033

**Keywords:** bimanual, context, imagery, movement, neural prosthetic, parietal cortex

## Abstract

Recent studies in posterior parietal cortex (PPC) have found multiple effectors and cognitive strategies represented within a shared neural substrate in a structure termed “partially mixed selectivity” (Zhang et al., 2017). In this study, we examine whether the structure of these representations is preserved across changes in task context and is thus a robust and generalizable property of the neural population. Specifically, we test whether the structure is conserved from an open-loop motor imagery task (training) to a closed-loop cortical control task (online), a change that has led to substantial changes in neural behavior in prior studies in motor cortex. Recording from a 4 × 4 mm electrode array implanted in PPC of a human tetraplegic patient participating in a brain–machine interface (BMI) clinical trial, we studied the representations of imagined/attempted movements of the left/right hand and compare their individual BMI control performance using a one-dimensional cursor control task. We found that the structure of the representations is largely maintained between training and online control. Our results demonstrate for the first time that the structure observed in the context of an open-loop motor imagery task is maintained and accessible in the context of closed-loop BMI control. These results indicate that it is possible to decode the mixed variables found from a small patch of cortex in PPC and use them individually for BMI control. Furthermore, they show that the structure of the mixed representations is maintained and robust across changes in task context.

## Significance Statement

Multiple effectors and cognitive strategies are represented within a small patch of human posterior parietal cortex (Zhang et al., 2017). However, it is unknown to what degree the structure of the representations is maintained across different task contexts. Here, we focus on the task contexts of brain–machine interface (BMI) training and online control, different contexts that have led to substantial changes in neural behavior in prior studies in motor cortex. We find that the structure of the representation of different movement conditions is largely maintained between the two contexts and that the different representations can all be separately decoded and used for online BMI control.

## Introduction

An important finding in systems neuroscience is that cortical neurons exhibit mixed selectivity (i.e., individual neurons are tuned to multiple variables in idiosyncratic ways). Early studies examining the representation of extrapersonal visual space in the posterior parietal cortex (PPC) of nonhuman primates (NHPs) showed that individual neurons, instead of having eye position-invariant receptive fields, combined retinotopic receptive fields with eye position signals (Andersen and Mountcastle, 1983). These two signals often interacted multiplicatively and were referred to as “gain fields” (Andersen et al., 1985). Neural networks trained to transform retinotopic receptive fields to craniotopic receptive fields also formed gain fields similar to the neural data (Zipser and Andersen, 1988). PPC neurons also mix head position (Brotchie et al., 1995) and vestibular signals (Snyder et al., 1998) for potential transformations to body and world coordinates. Recent studies found random mixing of modality (auditory and visual) in rat PPC (Raposo et al., 2014) as well as choice (sensory and rule) in NHP prefrontal cortex (PFC; Rigotti et al., 2013). Mixed selectivity allows a relatively small population of neurons to encode a large variety of variables (Fusi et al., 2016).

Our laboratory has observed a form of mixed selectivity within human PPC (Zhang et al., 2017), finding that cognitive strategy (imagine vs attempt) and body side (left vs right) variables were more overlapping in representation than the body part variable (hand vs shoulder). We termed this structure where some variables are more or less overlapping “partially mixed selectivity”.

In parallel, studies have found that neural representations in high-level brain areas such as PPC can vary with task context, such as the reward or value of an action (Platt and Glimcher, 1999; Iyer et al., 2010) or a pro-/anti-reach-mapping rule (Gail and Andersen, 2006; Westendorff et al., 2010). Another such context is open-loop motor imagery versus closed-loop cortical control of a brain–machine interface (BMI). Context here refers to prior information that changes the factors surrounding the movement but not the movement itself. Just as the value or mapping rule of an action might change planning and decision-making processes without altering the motor movements, real-time feedback during closed-loop control could do the same. Several studies have shown that the tuning of specific neurons can change from the initial training task (open-loop) to the online control task (closed-loop), although the underlying intentions are qualitatively unchanged in motor cortex (Taylor et al., 2002; Chase et al., 2009; Cunningham et al., 2011).

Nevertheless, BMI control based on representations measured during training is still possible, albeit frequently using a protocol that updates decoder parameters to account for changes in neural behavior under closed-loop control (Taylor et al., 2002; Santhanam et al., 2006; Aflalo et al., 2015). These studies focused on directional tuning properties used for BMI control with respect to a single effector or with different effectors in different sessions. To date, no studies have looked at how neural coding for additional movement attributes, such as cognitive strategy or representations of different effectors, compare between training and online control. Similarly, previous studies of mixed selectivity have focused on neural activity in the absence of any BMI online control, only examining the data that would be used to train a BMI decoder.

Thus, there remains an open question: how well is the structure of mixed representations conserved across different task contexts? In this study, we focus on the contexts of training and online control and test the BMI control performance of a C3/C4 tetraplegic participant in a 1D cursor control task, decoding from single-unit and multiunit activity recorded from a small 4 × 4 mm patch of the anterior intraparietal cortex (AIP). The participant controlled the cursor using imagined or attempted movements of the left (ipsilateral) or right (contralateral) hand. We find that all of the tested movement conditions remain differentially represented during online control, with the structure of the representations largely maintained. We also find that while attempted contralateral hand movements performed best, the effect was primarily driven by differences in the number of units tuned rather than differences in the consistency of the representations.

## Materials and Methods

### Subject details

Subject NS is a 59-year-old female tetraplegic 7 years postinjury at the time of the experiment, with a motor complete C3–C4 spinal lesion. She has no control or sensation of her hands, so attempted hand movements refer to trying to activate the muscles of the hand while imagined hand movements refer to mentally visualizing the movement (without any intended muscle activity or corresponding overt movement). Subject NS was right handed before injury. The study was approved by the California Institute of Technology, Casa Colina Centers for Rehabilitation, and the University of California, Los Angeles Institutional Review Boards. We obtained informed consent after explaining the objectives of the study and the possible risks involved. Subject NS was implanted with electrode arrays on August 26, 2014. The recordings for the primary study occurred in the interval between December 12, 2016, and January 30, 2017 (slightly over 2 years post-implantation). The recordings for the secondary study occurred in the interval between November 27, 2017, and December 22, 2017 (slightly over 3 years post-implantation).

### Experimental setup

Experimental sessions were performed at the Casa Colina Centers for Rehabilitation. Tasks were performed in a setup similar to that used in the study by Zhang et al. (2017), with NS seated in her motorized wheel chair in a dimly lit room. Tasks were presented on a 27 inch LCD monitor occupying ∼40º of visual angle, with stimulus presentation controlled using the Psychophysics Toolbox (David H. Brainard Laboratory, University of California, Santa Barbara, Santa Barbara, CA) and MATLAB. No fixation was required or enforced.

### Experimental design

We used a one-dimensional point-to-point control paradigm as the BMI control task. The participant was verbally instructed to control a cursor to move to the instructed target by “squeezing her hand to push the cursor upward, and relaxing her hand to let the cursor fall.” Movements were imagined or attempted squeezes of the left or right hand. The neural signals from the squeezes were decoded into an upward velocity signal such that the magnitude of the upward velocity was proportional to the magnitude of the neural responses (see Neural decoder for more details on the decoder specifications). Thus, NS could control the vertical position of a cursor by keeping her hand squeezed to push the cursor up and relaxed to move the cursor down, controlling the speed through her neural activity. The cursor was bounded by the edges of the screen, but targets were located such that overshooting was still possible. We used this relatively simple task to allow enough time for data collection for all four conditions within a single session.

The task was split into a training step (collecting data for the decoder training), and a testing step (evaluating the decoder’s performance during online control), with each step being a different task context. Separate training and testing steps were run for each of the four movement conditions (imagined/attempted left/right-hand squeezes). We verbally instructed NS on which movement condition to use before each training/online control run. For the training step, the cursor moved up and down automatically between two points, while NS was instructed to squeeze (as per the movement condition) in accordance with the direction of the movement of the cursor (i.e., squeezing when it was moving up, and relaxing when it was moving down). We used this task paradigm because it was more similar to an open-loop task where the movements are stereotyped and minimally affected by visual feedback (e.g., squeezes are similar in timing from trial to trial). At the same time, the paradigm still allows us to have more precise timing information on the movements to enable decoder training (e.g., the squeeze/release state based on cursor position relative to the target). Targets alternated between the two points, resulting in NS having to alternate between squeezes and releases consecutively. During the training step, movement of the cursor was completely decoupled from the participant’s brain activity; instead, movement of the cursor was determined by a linear quadratic regulator that was calibrated to perform point-to-point movements to the target in a naturalistic manner, reaching the target in ∼750 ms (Aflalo et al., 2015). Following movement, the cursor would rest on the target for the remaining trial duration (3.3 s total). We ran 32 trials per movement condition, with each trial composed of a squeeze-and-release phase.

For the testing step, NS was cued to move the cursor among three points oriented vertically in a center–out paradigm (as opposed to two points). This modified 1D task made control more difficult for NS than the previous two-point task (e.g., requiring more precise control to smoothly reach each of the three targets and minimize overshoot), and further emphasized a task context different from training. In each trial, NS was given 6 s to move to the target. For each point-to-point movement, if the allotted 6 s elapsed, then a secondary assist was activated, bringing the cursor to the target along the ideal trajectory computed above. This was done so that the initial distance between the starting position of the cursor and the target position was constant for each point-to-point movement and independent of the success/failure of the previous point-to-point movement. Between trials, the cursor was held constant at the center for 2 s without any target being presented [the intertrial interval (ITI)]. Note that NS’s motor intentions could affect the cursor position only when a target was presented. They could not affect the cursor position during the ITI, allowing her to relax during the ITI without it affecting the cursor position. Following the ITI, NS could then continue relaxing to allow the cursor to move downward, or squeeze her hand to make the cursor move upward. We ran 20 trials total for a single movement condition at a time. This equated to 40 point-to-point cursor movements (each trial split into a center-to-target and a target-to-center movement)—20 where the cursor was moving upward and 20 where the cursor was moving downward. The experiment was run on 8 separate days, with 593 units recorded in total (assuming independent populations between days, 74.13 ± 2.9 units/d). We tested all four movement conditions each day, with the order of the movement conditions changed each day to avoid potential order effects that could cause performance differences (e.g., performance being better for early runs than late runs).

In the primary task design, each training run for a condition was immediately followed by online control for the same condition (see Fig. 4*E*). This prevented us from disambiguating whether some results were driven by the conditions being closer in time or by the conditions being more similar/matched. For example, recording nonstationarities (with units appearing or disappearing over the course of a session) or neural responses dependent on the local history of task conditions could introduce a temporal component to the neural data and cause consecutive pairs of runs to have more similar neural representations than pairs further apart in time. This would make our result that the representations are preserved from training to online control (see Fig. 4*A*,*B*) ambiguous. The analysis for that result compares different runs of the same condition but differing by context [e.g., attempted right-hand (ARH) training and ARH online control].

As a control for temporal order, we collected data using a secondary task to determine how well representations are maintained across a longer interval of time. This version was identical to the primary task but with the order of the runs changed such that training and online control runs of the same condition were no longer always temporally adjacent (see Fig. 4*F*, example blocks). For example, in each block of four runs, we interleaved two movement conditions, A and B, in the order: training for condition A, training for condition B, online control for condition B, online control for condition A. This experiment was run on 4 separate days, with two blocks of four runs per day (eight runs total), and 220 units recorded in total (assuming independent populations between days).

### Signal recording procedures

Two 96-channel Neuroport arrays (models #4382 and #4383, Blackrock Microsystems) were implanted in the putative homologs of area AIP and Brodmann’s area 5d. Preoperative fMRI was used to identify array implant location (Aflalo et al., 2015). Only data recorded from the array implanted in AIP (at Talairach coordinate: −36 lateral, 48 posterior, 53 superior) were analyzed and presented here. A Neuroport neural signal processor (NSP) amplified, digitized, and recorded the neural activity. The Neuroport System (composed of both the NSP and the arrays) has Food and Drug Administration (FDA) clearance for acute recordings over a duration <30 days. For this brain–machine interface clinical trial, we have received FDA investigational device exemption (IDE) clearance (IDE #G120096, G120287) to implant and record from PPC past the 30 d limit.

During recording, full-bandwidth signals were sampled at 30 kHz in the Central software suite (Blackrock Microsystems) and were high-pass filtered (250 Hz cutoff). We used −3.5 times the root mean square as the threshold for action potential detection. Each wave form was composed of 10 samples before triggering and 38 samples after, with a total duration of 1.6 ms. Both sorted units and threshold crossings without sorting on the AIP array (hereafter referred to altogether as “units”) were recorded and analyzed. Units were sorted by hand at the session before any data collection using the Central software suite (Blackrock Microsystems), with only high-quality, easily isolatable, single units and multiunits being identified and sorted (26.6 ± 2.58 sorted units/d). We wanted to analyze the neural data recorded during a real-time online session as opposed to an offline analysis with intensive spike sorting. This approach better emulates the conditions of a real-time BMI, so we purposefully did not perform any additional offline sorting.

### Decoding procedures

We used a continuous decoder fit on the vertical velocity of the cursor as a linear function of the neural population as a whole. The position of the cursor was bounded by the edges of the screen, but targets were positioned such that it was still possible for the cursor to overshoot. Note that the decoder used for control was trained on only one strategy–effector combination at a time, differentiating between the squeeze state and the release state for that particular condition.

For features, the velocity decoder used the *z*-scored firing rates of the units. Only units with a minimum raw firing rate of at least 1 Hz were included (on average, 96.3 ± 0.2% of all units across all sessions). Firing rates were sampled with a 50 ms sampling period and then smoothed by a causal exponential filter with a 1.5 s duration (length of 30 samples) and a smoothing factor of 0.75 to filter out high-frequency noise.

For the regression, we used the lasso function in MATLAB, which implements elastic net regularization. This method tries to minimize the number of terms in the model based on an elastic net mixing value that trades off between lasso and ridge regression (a mixing value of 1 is lasso regression, while a mixing value of 0 is ridge regression). We used an elastic net mixing value of 0.05 for the model, with the exact lambda (regularization coefficient) determined through cross-validation (across 15 values of lambda).

### Statistical analysis

Comparisons between pairs or multiple distributions were performed using nonparametric one-way ANOVA (Kruskal–Wallis test), grouping by the different movement conditions being compared (either all four or in the pairwise case two at a time). Samples were the values computed on a per day basis (see Figs. 3, 4*A*), a per trial basis (see Fig. 7*A*,*B*), a per run basis (see Figs. 4*C*,*D*, 7*C*), or a per unit basis (see Figs. 2, 6, 7*D*).

Values shown in figures were computed from datasets after pooling across all days. Error bars indicate bootstrapped confidence intervals on the pooled data. All such confidence intervals were computed with 2000 bootstrap data samples.

All analyses were performed using MATLAB 2017a.

### Unit selection

Units were pooled across days assuming independent populations. Only units with mean firing rates >0.5 Hz and with a signal-to-noise ratio >0.5 were included in the analyses to limit low firing rate and noise effects.

### Linear model analysis for neural activity characterization

For each individual unit, we fit a linear model to the firing rate of the unit when the hand was released and squeezed. This was done for each movement condition separately (one model fit per condition, eight models total). The hand was considered to be in a squeeze or release state based on the position of the target relative to the cursor, and which action NS would need to perform to successfully complete the trial. For the time window of neural activity, we wanted to minimize potential feedback corrections and isolate the movement intention signals. Based on single-unit and multiunit event-related averages from this and past studies (Zhang et al., 2017), we chose a window of 500–1500 ms after cue onset to isolate the majority of the neural modulatory activity. The significance value of the fit (*p* value of the *t* statistic for the beta coefficients) was used to determine tuning to the corresponding condition [significant if *p* < 0.05, false discovery rate (FDR) corrected]. Because the linear model fits the firing rate of a unit as a function of whether the hand was in a squeeze or release state, a unit is thus considered “tuned” if its firing rate significantly modulates between when the hand is squeezed and released. We also analyzed the *R*^2^ value of the fit as a measure of the reliability of tuning (i.e., consistency in terms of trial-to-trial variability). Additionally, we analyzed the beta coefficient of the model and its cross-validated SE as another measure of tuning magnitude (i.e., the magnitude of the firing rate modulation) and reliability.

### Degree of specificity

Before assessing any possible performance differences between the movement conditions, we needed to verify that the conditions were differentially represented in our neural population. To do this, we performed a degree of specificity analysis, characterizing the degree to which units were specific to different levels of a variable in training and online control. We first looked at one level of a variable (e.g., the right hand) and computed the degree of specificity to the levels of the other variable (e.g., specificity to imagine and attempt). The degree of specificity was computed by taking the difference of the absolute values of the relevant beta coefficients (those associated with the two levels being compared) normalized by the sum of the absolute values of the beta coefficients. For example, to compute the degree of specificity to imagine and attempt with the right hand, the equation would be as follows:

$$Degree\;of\;Specificity=\frac{\left|\beta_{ARH}|-|\beta_{IRH}\right|}{\left|\beta_{ARH}|+|\beta_{IRH}\right|},$$

where β is the beta coefficients from the linear model fit above for the associated movement condition divided by the SE of the beta estimate. This degree of specificity analysis was performed separately for the training data and the online control data. We only included units tuned to at least one of the conditions being compared (*p* < 0.05, FDR corrected). For each distribution, we performed a two-sided sign test to determine whether the medians of the distributions were significantly different from 0 (i.e., whether they were biased to one variable over another).

We also wished to show how well preferences were maintained on a per unit basis across training and online control. As with the degree of specificity, we first looked at one level of a variable (e.g., the right hand), and computed the “condition preference” to the levels of the other variable (e.g., imagine and attempt). We quantified the condition preferences of each unit as the differences between beta coefficients normalized by their SEs. This is the same computation as the degree of specificity, but without the normalization term in the denominator, a choice made given that we wanted to look at individual unit changes from training to open loop and that these changes are more interpretable at their natural scale where normalization of small-amplitude differences cannot obscure the magnitude of condition differences. Condition preference values are displayed as a paired-point plot showing the values for training and online control with a connecting line for each unit. The full distribution of the condition preferences are shown as a violin plot. For each distribution, we performed a two-sided sign test to determine whether the medians of the distributions were significantly different from 0 (i.e., if they were biased to one variable over another). Finally, we computed the change in condition preference from training to online control. To test whether the condition specificity was preserved, we compared the fiducial values of the distances (e.g., between the same unit) with a null model in which differences between training and online control were computed after shuffling unit identity. These two distributions (fiducial differences vs shuffled) were compared using a two-sample *F* test for equal variances.

### Correlation between neural representations

In addition to the single-unit and multiunit measures of how well representations were maintained, we also wanted a population measure of the similarity between representations during training and online control. Using correlation as a measure of similarity, we directly looked at the similarity of the neural representations for each condition separately. We used correlation over other distance measures (e.g., Euclidean or Mahalanobis distance) because correlation gives a normalized value of the similarity between the representations and is invariant to gross baseline changes across the entire population.

The neural representations were the vector of normalized beta coefficients (one element for each unit). The normalized beta coefficients are the beta coefficients (from the linear models above) normalized by their 95% confidence intervals and thus are a trial average measure of the activity of each unit weighted by its trial-to-trial variability.

### Comparison of representations between training and online control

The above analyses treat each condition separately, looking at how well representations were maintained from training to online control. We also wanted to test how well the structure of the representations of the movement conditions as a whole was preserved going from training to online control (i.e., the relationships between the representations). We performed a cross-decoder analysis, training a linear classifier (linear discriminant analysis, equal diagonal covariance matrices for each condition) on just the training data (combined across condition) to classify the four movement conditions, and then tested the ability of the classifier to generalize to the online control data. The classifier used the modulation of the firing rates of the units from the release state to the squeeze state as features. The cross-validated performance of the classifier on training data was also computed for comparison. Also, a second classifier was trained on the online control data and tested on the training data (testing generalization from online control representations to training representations). We performed this cross-decoding analysis independently for each of the experimental sessions, resulting in a distribution of scores for the cross-validation/generalization scores of each classifier. For each score distribution, we used a one-sided Wilcoxon signed rank test to determine whether the scores were significantly above a chance prediction score of 1 of 4 (0.25, *p* < 0.05).

To look at how well the classifiers performed with each condition during generalization, we also computed the confusion matrices for each of the generalization scores. For each trial, we recorded the true condition type as well as the condition type predicted by the classifier. These true condition and predicted condition pairs were counted up and tabulated into a matrix form and then normalized by the number of trials per condition, resulting in the confusion matrix values shown in Figure 4*B*.

### Analysis to control for order effects

To control for the possibility that the results of the above analysis were driven by the temporal adjacency of different conditions, we performed an additional control analysis. Looking at the primary task dataset, we compared the similarity between neural responses from training to online control when the conditions were matched (and therefore adjacent due to the design of the primary task) against the similarity when the conditions were mismatched but still adjacent in time. For example, consider the set of runs in the sequence depicted in Figure 4*E*. We compared the similarity in the responses of consecutive pairs with matched conditions (pairs marked in blue) against the consecutive pairs with mismatched conditions (pairs marked in red). We measured similarity as the correlation between the beta coefficients of the conditions. The distributions of the correlation values (computed on a per-pair basis) were then compared between the two groups (matched vs mismatched) using a nonparametric one-way ANOVA (Kruskal–Wallis test).

### Analysis of temporal effects

We also wanted to examine whether representations between training and online control could be maintained across extended periods of time. Analyzing data from the secondary experimental design, we directly compared different pairs of runs within each block of four runs (see Experimental Paradigm; see Fig. 4*F*). We compared the correlations between the second and third runs (pairs marked in blue, matched by condition and closer together in time) against the correlations between the first and last runs (pairs marked in yellow, matched by condition but farther apart in time). We could also compare the above correlations with the pairs of training and online control runs with mismatched conditions by computing the correlations between the first and third runs (pairs marked in red, mismatched by condition). The distributions of the correlation values (computed on a per-pair basis) were again compared with a nonparametric one-way ANOVA (Kruskal–Wallis test).

### Neural performance

We used the performance of individual units during online control as a continuous measure of how well units maintained their tuning. We used this measure instead of simply looking at how many units maintained their tuning between training and online control. One drawback of only using tuning is that it makes a binary determination of whether or not a unit is tuned. It is possible for a unit to be classified as tuned during both training and online control but with different strengths (i.e., different beta coefficient magnitudes). For example, a unit could be significantly modulated by a condition during both training and online control but with significantly different firing rates.

To compute the performance of each individual unit, we looked at the direction of the influence of the unit on the cursor within a time window (e.g., the window of 500 –1500 ms used in the above linear analysis). For the given time window, the neural activity was projected through the corresponding decoder weight (from a decoder trained on the training data) into an influence acting on the cursor. The sign (direction) of the average influence across the time window indicated whether the unit was pushing the cursor up or down on average during that interval. We then compared the direction of this influence to the direction the cursor would need to move in to reach the target. In other words, we looked at whether the unit was pushing the cursor in the correct direction on average. This influence was computed on a trial-by-trial basis for the selected time window. The neural performance of an individual unit was then defined as the fraction of trials where the unit was pushing the cursor in the correct direction.

When aggregating these unit measures, we used a weighted average of neural performance values, with weights taken from the corresponding population decoder used for online control. This allows us to directly measure the generalization performance of each unit in the context of its effect on online control performance. In other words, the weighted average is reflective of the performance of a decoder trained on the units being pooled together. While comparing firing rates between training and online control would also be informative of how well units maintained their tuning, it would not account for the actual influence of the units on the online control. Note that for the decoder weights we used the weights from the population decoder as opposed to the single-unit beta coefficients.

### Behavioral performance metrics

We used two metrics to assess behavioral control performance. The first metric was simply the number of successful trials divided by the total number of trials (success rate). A trial was considered successful if the cursor reached the designated target and was held there for 1 s within the allotted 6 s (under the control of NS). The second metric was the time required to reach success (not including the 1 s hold time), looking only at successful trials.

To get a sense of what constituted a “good” success rate, we used real neural data to simulate a bound on chance performance. For each online control run, we simulated trials by randomly selecting a mock target, randomly selecting a 6 s segment of neural data, applying the corresponding decoder, and simulating the trajectory of the cursor. We then marked the simulated trial as a “success” if the simulated trajectory reached the mock target and was held there for 1 s (the same criteria as was used on the real online control trials). The mock targets were either the true target for a trial or the target equidistant from the starting position but in the opposite direction as the true target (e.g., flipping the direction) so that the travel distances would be the same. The cursor bounds keeping the cursor on screen were also translated as needed so that the simulated trials had bounds symmetrical to the real trial. For example, consider a true trial where the cursor moved from the top target down to the center and with a boundary directly above the starting position, preventing it from moving upward too much. If the mock target was above the starting position, then the boundary would be shifted so that there was a boundary directly below the starting position, preventing it from moving downward too much. This was done so that the effects of the boundaries on performance would be replicated in the simulation as well. This simulation method allows us to determine whether our observed control performance was significant (i.e., whether NS had control).

## Results

**Figure 1.**
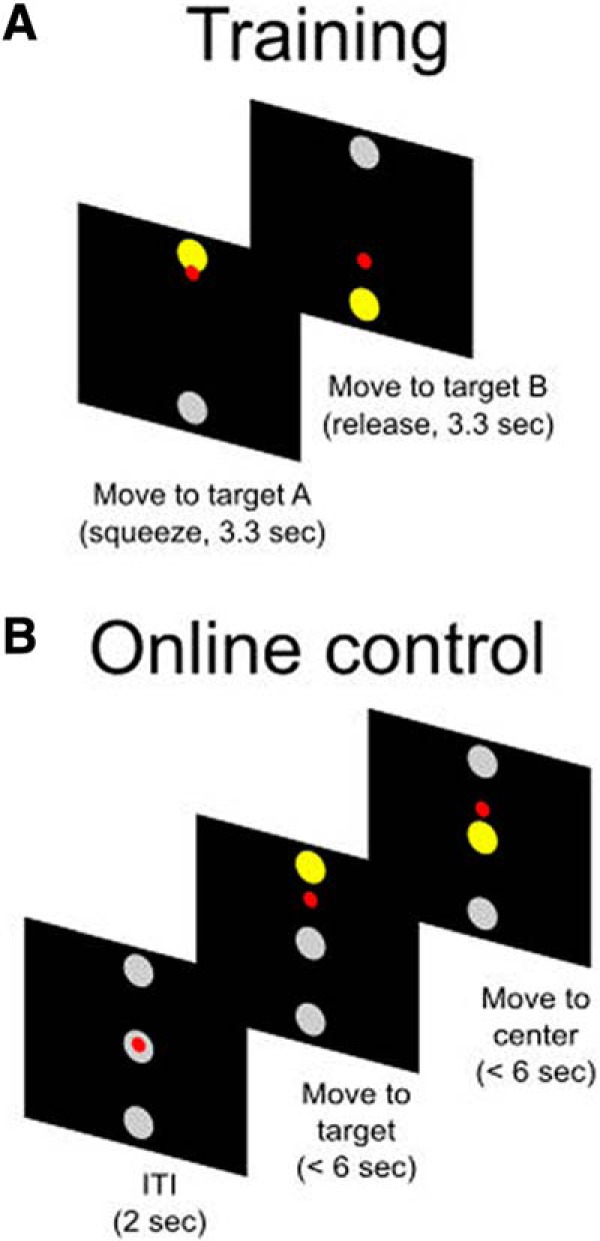
Experimental paradigm. ***A***, Training task. The small red circle is the cursor, the gray circles are the possible targets, and the yellow circle is the target for the specific trial. ***B***, Online control task.

As detailed in the Materials and Methods section, we recorded from AIP of a female, C3/C4 tetraplegic participant (NS) and compared neural responses of four movement conditions during the calibration (“training”) and online control steps of a 1D BMI control task (Fig. 1*A*,*B*). The four movement conditions tested and used for control were as follows: (ARH) movements, imagined right-hand (IRH) movements, attempted left-hand (ALH) movements, and imagined left-hand (ILH) movements.

We first investigated how similarly the movement conditions were represented between training and online control, as well as the degree to which the structure of these representations (i.e., relationship between the representations) was maintained across the two contexts. We then examined whether all the tested movement conditions are feasible for control and the factors involved in any performance differences between the movement conditions, results that are particularly pertinent to the contexts of BMI control.

### Representations during training and online control contexts

**Figure 2.**
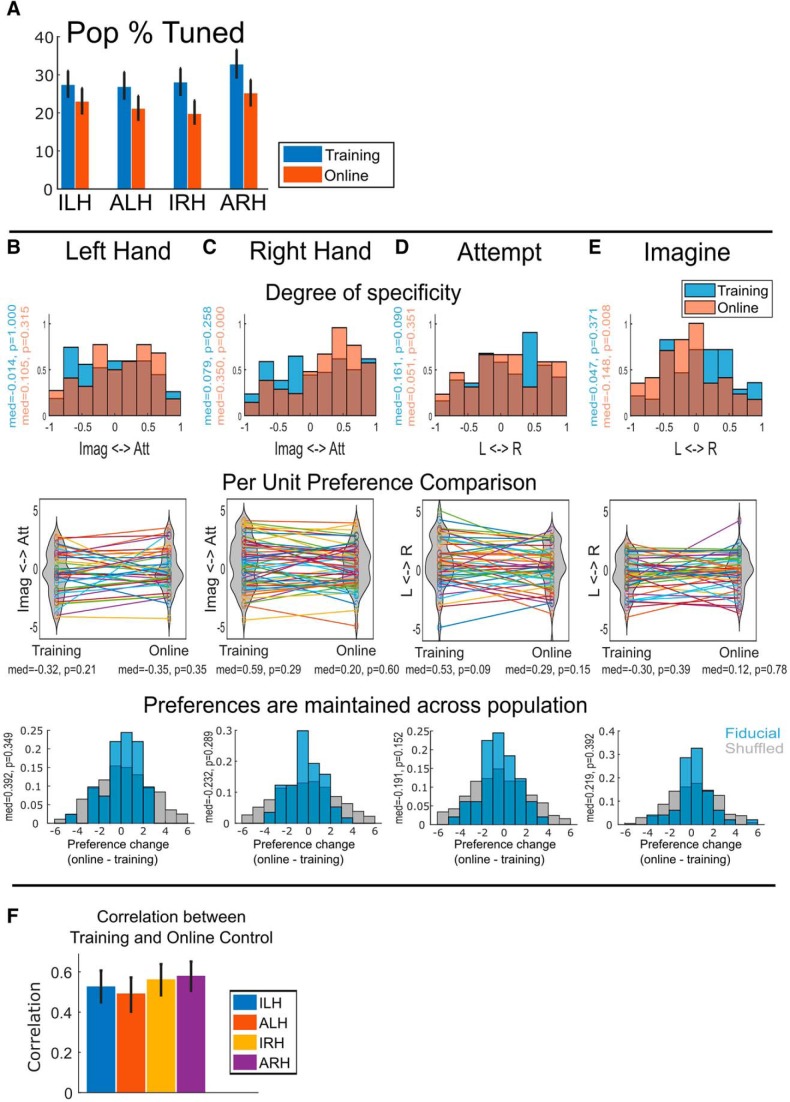
Tuning of the population to the conditions. ***A***, Percentage of units tuned to each movement condition (bootstrap 95% CI, *p* < 0.05, uncorrected). ***B***, Top row, Degree of specificity showing the distribution of how much units exclusively code ILH or ALH. Distribution during training shown in blue and distribution during online control shown in orange. For each distribution, the median and the probability the median is different from 0 (two-sided sign test) are shown in their corresponding colors. Middle row, Paired point plot showing how condition preferences for individual units changed from training to online control. Distribution during training and online control shown as a violin plot. For each distribution, the median and the probability the median is different from 0 (two-sided sign test) are shown underneath their corresponding x-label. Bottom row, Distributions showing change in preference values showing in the middle row between training and online control while preserving unit identity (fiducial, blue) and when shuffling unit identity (shuffled, gray). For each fiducial distribution, the median and the probability the median is different from 0 (two-sided sign test) are shown underneath their corresponding x-label. ***C***, Similar to ***B***, but for IRH and ARH. ***D***, Similar to ***B*** but for ALH and ARH. ***E***, Similar to ***B*** but for IRH and ILH. ***F***, Correlation between movement representations during training and online control (bootstrap 95% CI).

We first wanted to verify that the four movement conditions were represented in the recorded population, during both training and online control, by looking at the percentage of the population tuned to each condition. For each unit, movement condition, and context separately, we fit a linear model to the firing rate modulation of the unit between the “release” to “squeeze” hand states (see Materials and Methods for more details). A unit was considered tuned if the beta coefficient of the model was significantly different from zero (*p* < 0.05, uncorrected). A significant fraction of the population was tuned to each of the conditions (Fig. 2*A*), indicating that they were indeed represented.

We used a specificity analysis to examine the degree of overlap between the populations representing each condition (i.e., the degree to which the populations had shared/distinct subpopulations of units). If populations were nonoverlapping, we would expect to see most units having high specificity values. On the other hand, if populations were overlapping, the distributions would have lower specificity values (clustered around 0). Figure 2*B–E* (top row) shows the distribution of the specificity values focusing within one level of a variable at a time. For example, Figure 2*C* (top row) looks at the degree of specificity of the units to attempt and imagine only for conditions involving the right hand. In Figure 2*C* (top row), a value of 1 would correspond to a unit activated only by attempt and not at all for imagination, a value of −1 would correspond to a unit activated only by imagination and not attempt, and a value of 0 would correspond to a unit activated similarly by both. Units not significantly tuned to either were excluded from the analysis. We computed the distributions separately for the training data (Fig. 2*C*, blue) and the online control data (Fig. 2*C*, orange). All computed distributions indicated partially overlapping populations, with some units highly specific to a condition and other units equally responsive to both.

In Figure 2*B–E* (middle row), we show how preferences of individual units are preserved from training to online control in a paired-point plot. Each point shows the preference value for training and online control, and a connecting line enables tracking how the preference of the unit changes across contexts. Figure 2*B–E* (bottom row) quantifies these changes by plotting a histogram of the online values subtracted from the training values. To quantify whether preference values tend to be similar or change across contexts, we compared the fiducial distribution of changes against a distribution constructed by subtracting online from training data while scrambling unit IDs. In each case, the fiducial distribution was more narrowly distributed than the shuffled distributions (two-sample *F* test for equal variances, *p* < 0.05). Together, these results show that, at the individual unit level, preferences tend to be maintained, although changes, sometimes substantial, can also be found.

Given that all four tested movement conditions were represented in different but partially overlapping populations, we first wanted to see how well the representations were maintained across the population. Using correlation as a measure of similarity, we correlated the neural representations of each condition during training to its corresponding representation during online control (see Materials and Methods for more details). There were no significant differences between conditions going from training to online control (Fig. 2*F*; χ^2^(3, *N* = 32) = 6.59, *p* = 0.087, Kruskal–Wallis test).

**Figure 3.**
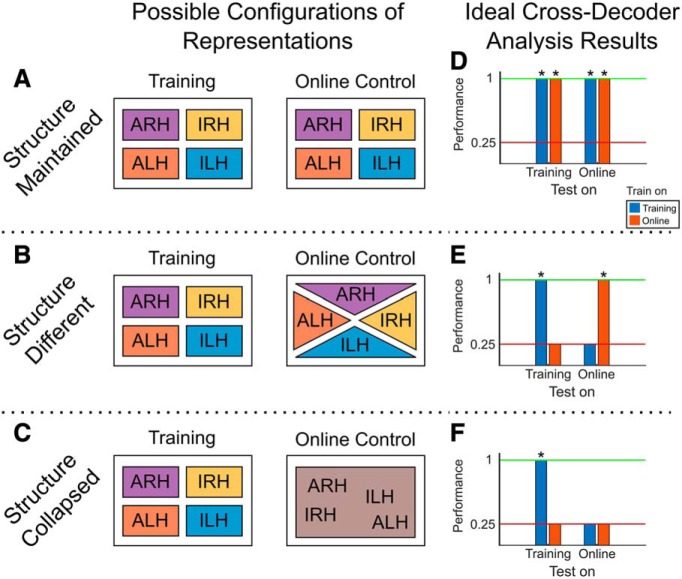
Possible configurations of representations and corresponding expected analysis results. ***A–C***, Schematics for different possibilities in how the structure of the representations compares between training and online control. ***A***, Schematic for the “structure maintained” case where the structure is consistent between training (left) and online control (right). Representations of the four movement conditions are separable during both training and online control, and in the same structure (i.e., the same configuration, as represented by the consistent placement of the conditions). ***B***, Schematic for the “structure different” case where the movement conditions are separable during both training (left) and online control (right) but with different structures (i.e., different configurations). ***C***, Schematic for the “structure collapsed” case where the movement conditions are separable during training only (left) and collapse into a single representation (as represented by the conditions being no longer separable in the online control case, right). ***D–F***, Ideal expected result from cross-decoding analyses if the data follow the different schematics in Figure 3*A–C*. See Results for detailed explanation of colors and bars. Red lines represent chance performance (0.25). Performances significantly above chance are marked with an asterisk. ***D***, Ideal expected result in the “structure maintained” case of Figure 3*A*. ***E***, Ideal expected result in the structure different case. ***F***, Ideal expected result in the structure collapsed case.

The above analyses in Figure 2 suggest that the representations of all four conditions are largely preserved between training and online control. The comparable level of maintenance between each of the conditions also suggests that the structure of the representations itself is maintained. However, this is not an obvious result. The above analyses treat each condition separately and do not look at how the relationship between the conditions changes between training and online control. In Figure 3*A–C*, we show the following three main possible configurations of the structure of the representations going from training to online control: (1) the structure of the representations is maintained and consistent between training and online control, as the above results seem to suggest (structure maintained; Fig. 3*A*); (2) all four conditions are represented during online control but in a different structure than during training (structure different; Fig. 3*B*); and (3) the representations of the four conditions collapse into a single representation that is invariant to which of the four conditions is being used, such as in a pure intention or goal signal (structure collapsed; Fig. 3*C*).

To more directly adjudicate among the three configurations, we performed a cross-decoding analysis to test how well the representations of the four conditions generalize across the contexts (i.e., across training and online control). We trained a linear classifier on the training data to classify among the four movement conditions and tested it on the training data (cross-validated performance) and the online control data. Conversely, we also trained a classifier on the online control data and tested it on the online control data (cross-validated performance) and the training data (see Materials and Methods for more details).

The results of such an analysis can directly clarify which of the above three configurations fits our data best. Idealized example results corresponding to each of the three configurations are shown in Figure 3*D–F*. If the structure is maintained (Fig. 3*A*), we would expect results similar to those in Figure 3*D*. The training classifier in this case performs well within the trained-on training data (Fig. 3*D*, left blue bar, cross-validated performance), as well as with the not-trained-on online control data (Fig. 3*D*, right blue bar). Similarly, the online control classifier performs well both with the trained-on online control data (Fig. 3*D*, right red bar; cross-validated performance) and with the not-trained-on training dataset (Fig. 2*F*, left red bar). This pattern of performance indicates that the four conditions are differently represented in both training and online control and that the structure of these representations is similar between the two contexts. On the other hand, if the structure is different (Fig. 3*B*), then we would expect results similar to those in Figure 3*E*, where both cross-validated performances are significant, but generalization performance is only at chance level. Finally, if the structure collapsed during online control (Fig. 3*C*), we would still expect cross-validated performance for the training classifier to be significant and generalization performance to be at chance level, but we would also expect cross-validated performance for the online control classifier to be at chance level, indicating that the representations are not differently represented during online control (Fig. 3*F*).

**Figure 4.**
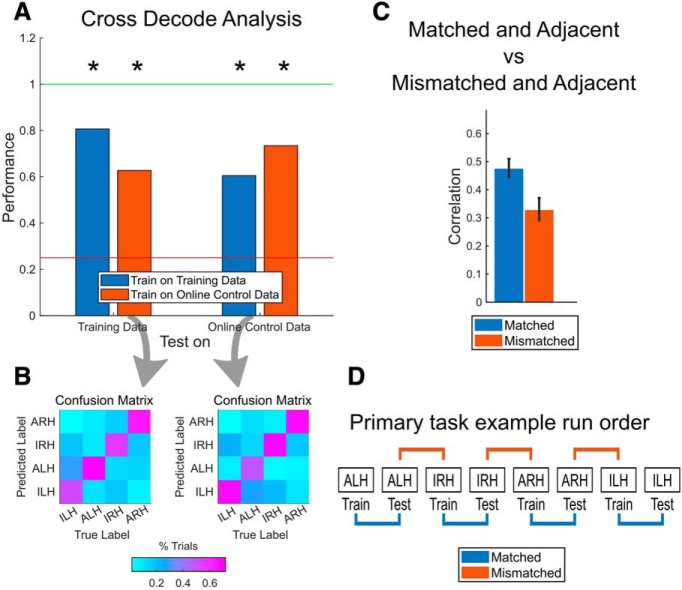
Maintenance of the structure of representations. ***A***, Results of the cross-decoding analysis performed on our data, presented as in Figure 3*D–F*. Performances significantly above chance are marked with an asterisk (one-sided Wilcoxon signed rank test, *p* < 0.05; see Materials and Methods for more details). ***B***, Confusion matrices showing classifier predictions when generalizing from one context to the other, shown as the percentage of trials per condition. Columns are the true condition labels, and rows are the predicted labels. Left matrix corresponds to the classifier trained on the online control data and tested on the training data (Fig. 4*A*, left red bar). Right matrix corresponds to the classifier trained on the training data and tested on the online control data (Fig. 4*A*, right blue bar). ***C***, Correlation between neural representations of pairs of runs where the runs were adjacent in time and matched in condition (blue), compared with the correlation between pairs adjacent in time mismatched in condition (red). Error bars are 95% bootstrapped confidence intervals. See Materials and Methods for more details. ***D***, Example set of runs from a single session for the primary task paradigm (see Materials and Methods). Pairs marked in blue are matched by condition and are adjacent in time while pairs marked in red are mismatched in condition but still adjacent in time.

We found that the classifiers generalized from training to online control (and vice versa; Fig. 4*A*). This suggests that the structure of the representations of the four movement conditions is maintained and meaningful across the two contexts (structure maintained; Fig. 3*A*). The confusion matrices show that the representations of all four conditions tended to generalize equally well (Fig. 4*B*).

In our experimental procedure, online control runs always occurred directly after the training run of the corresponding condition. As a result, the above cross-decoding generalization could simply be due to different contexts of the same condition type (e.g., ARH training and ARH online control) always being temporally adjacent to each other. To take one example, nonstationarity in the structure of representations on a time-scale longer than adjacent runs could account for temporally adjacent epochs of neural data having more similar representations than temporally distant epochs.

To control for this possibility, we compared the correlations of the neural representations between training and online control (Fig. 4*D*, pairs marked in blue; temporally adjacent, “matched” by condition), against the correlations between the other temporally adjacent condition combinations (pairs marked in red, temporally adjacent, but “mismatched” by condition). In other words, we compared the similarity of the neural representations between each training run and its following online control run (matched and adjacent) against the similarity of each training run and its preceding online control run (mismatched and adjacent). Despite all examined pairs of datasets being temporally adjacent, the correlations between pairs matched by condition were significantly higher than those not matched by condition (Fig. 4*C*; χ^2^(1, *N* = 56) = 20.88, *p* = 4.89e-6, Kruskal–Wallis test).

**Figure 5.**
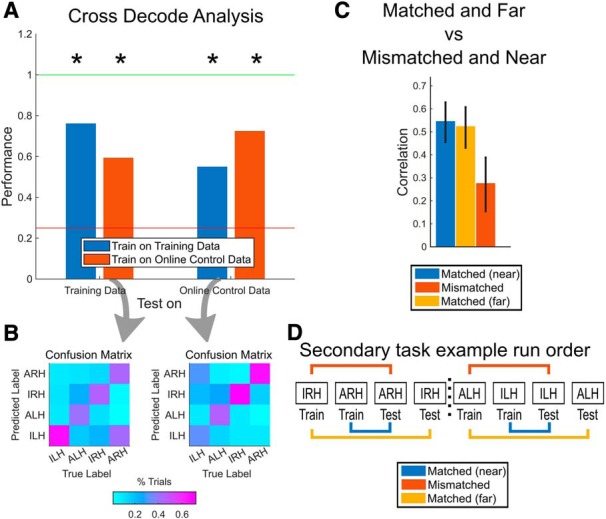
Maintenance of structure of representations preserved for altered timing of condition blocks. ***A***, Results of the cross-decoding analysis performed on additional data in which training and online test runs were collected with modified ordering of task conditions, presented as in Figure 4. Performances significantly above chance are marked with an asterisk (one-sided Wilcoxon signed rank test, *p* < 0.05; see Materials and Methods for more details). ***B***, Confusion matrices showing classifier predictions when generalizing from one context to the other, shown as the percentage of trials per condition. Columns are the true condition labels, and rows are the predicted labels. Left matrix corresponds to the classifier trained on the online control data and tested on the training data (Fig. 4*A*, left red bar). Right matrix corresponds to the classifier trained on the training data and tested on the online control data (Fig. 4*A*, right blue bar). ***C***, Correlation between neural representations of pairs of runs matched by condition but farther apart in time (blue) compared to pairs mismatched by condition but closer together in time (red). Error bars are 95% bootstrapped confidence intervals. See Materials and Methods for more details. ***D***, Example of two blocks of runs (four runs per block) for the secondary task paradigm used to control for an order effect (see Materials and Methods). Pairs marked in blue are matched by condition and are close in time, pairs in yellow are matched by condition but are farther apart in time, while pairs in red are mismatched by condition but are closer in time.

We were also interested in whether the structure of the representations could be maintained after extended periods of time. We collected several secondary datasets where we used the following order in each “block” of runs, training for condition A, training for condition B, online control for condition B, and online control for condition A—where the conditions A and B were each selected from the four movement conditions (Fig. 5*D*, example blocks of runs). This ordering allowed us to directly compare how well representations were maintained across training and online control runs closer together in time with how well they were maintained across runs farther apart in time. Importantly, we found that the classifiers generalized from training to online control (and vice versa; Fig. 5*A*,*B*) similar to the initial experimental configuration (Fig. 4*A*,*B*). Further, We compared the correlations between the neural representations (beta values) of the first and last runs of each block (pairs marked in yellow in Fig. 5*D*, same conditions, but farther apart in time) against the correlations between the neural representations of the second and third runs (pairs marked in blue, same conditions, but closer together in time). The correlation values were not significantly different across the population (Fig. 5*C*; χ^2^(1, *N* = 16) = 0.044, *p* = 0.83, Kruskal–Wallis test), suggesting that the representations are similarly well maintained for runs nearer in time as for runs farther apart in time.

In addition to comparing how well representations were maintained across training and online control for matched conditions, we also examined how well they were maintained for mismatched conditions. The neural representations were more similar when the conditions were matching, rather than when the conditions were mismatched, both when comparing against the condition pairs nearer in time (χ^2^(1, *N* = 16) = 8.65, *p* = 0.0033, Kruskal–Wallis test) and farther apart in time (χ^2^(1, *N* = 16) = 9.28, *p* = 0.0023, Kruskal–Wallis test). Altogether, the results of Figures 4*C* and 5*C* suggest that the “structure maintained” configuration found in Figures 4*A* and 5*A* is not a result of the fact that the conditions were temporally adjacent. Rather, the result was driven by the actual matching of the conditions. Furthermore, the representations are maintained similarly well even after an extended period of time with other conditions being tested.

The above results show that the structure of the representations of the different movements is relatively consistent between training and online control, with significant generalization in the organization from one context to the other. However, the generalization is not perfect, with the generalization performance still lower than the cross-validated performance. Figure 4*B* already shows that the representations generalize equally well for each of the movement conditions, albeit imperfectly, meaning that there is no single movement condition that causes the generalization performance to drop.

The drop in generalization performance could also be due to a specific subset of units generalizing poorly, rather than all units (regardless of tuning preference) generalizing imperfectly. Thus, we next asked whether there was a systematic difference in how well specific units generalized based on their tuning preference. For example, do imagine-specific and attempt-specific units both maintain their specificity equally well or does one type generalize to online control better?

**Figure 6.**
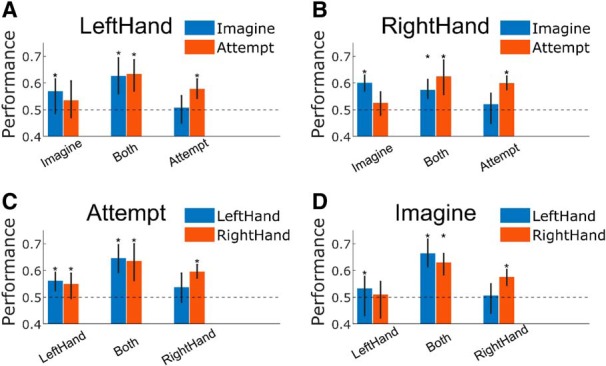
Maintenance of representations split by tuning preference. ***A***, Average single-unit performance (weighted by the corresponding decoder weights) for imagined/attempted left-handed movements (bootstrap 95% CI). Units are grouped by tuning only to attempted movements, tuning only to imagined movements, and tuning to both. Performance was evaluated for imagined left-hand movements (blue bars) and attempted left-hand movements (red bars). Performances significantly above chance (one-sided sign test, *p* < 0.05, FDR corrected) are marked with an asterisk, and chance performance is marked by the dashed line. ***B***, Similar to ***A*** but for right-handed movements. ***C***, Average single-unit performance (weighted by the corresponding decoder weights) for left/right-handed movements using the attempt strategy. Units are grouped by specificity of tuning to the left or right hand, with performance evaluated during left- and right-handed movements (blue and red bars, respectively). Significant performances are marked. ***D***, Similar to ***C*** but for movements using the imagine strategy.

To answer this question, we focused within one level of a variable at a time (e.g., the right hand), categorized units by their specificity to the levels of the other variable during training (e.g., only tuned to attempt, only tuned to imagine, or tuned to both), and then assessed their performance during online control (Fig. 6; see Neural performance in Materials and Methods for more details). In general, units that were specific to one condition maintained their specificity in terms of performance during online control. This was true for each of the four conditions. Likewise, units that were nonspecific between the compared conditions (“both”) performed equally well with either condition, maintaining their nonspecificity. For example, units that were tuned to ARH and not IRH movement during training performed above chance with ARH and not with IRH and vice versa (Fig. 6*B*). Similarly, units that were responsive to both ARH and IRH performed comparably well with both ARH and IRH during online control (Fig. 6*B*). These results indicate that the tuning preference of a specific unit does not affect how well it will generalize from training to online control and that there is no specific functional variable that generalizes better than another. These results also further emphasize that the unit-tuning preferences observed during training are largely maintained and meaningful during online control, consistent with our population results above.

### Comparison of movement conditions and online control performance

We also focused on whether all the tested movement conditions are feasible for online BMI control, the degree to which performance differs between the movement conditions, and the possible causes of the performance differences. For the context of BMI control, the ability to use the multiple movement conditions and decode the many variables is particularly important.

**Figure 7.**
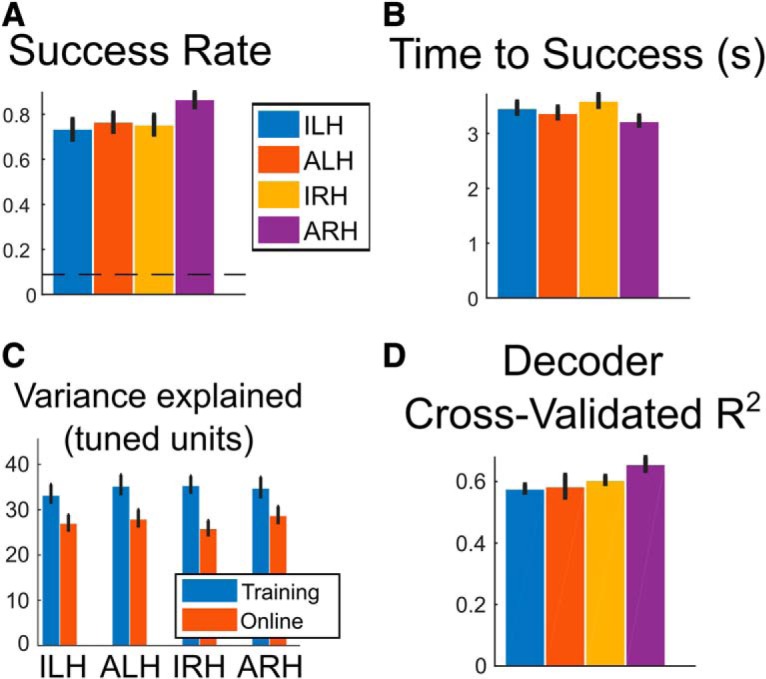
Online control performance. ***A***, Performance of each movement condition, measured as the fraction of successful trials (bootstrap 95% CI). Dashed line indicates simulated chance performance (see Materials and Methods). ***B***, Performance of each movement condition, measured as the mean duration of successful trials (bootstrap 95% CI). ***C***, Mean *R*^2^ of units tuned to each movement condition from Figure 2*A* (bootstrap 95% CI). ***D***, Cross-validated *R*^2^ of the decoder used for online control, trained on the training data for each condition (bootstrap 95% CI). Cross-validated *R*^2^ was computed for each condition and session separately.

We compared the performance of the movement conditions individually, looking at both trial success rate (the fraction of trials where NS successfully moved the cursor to the target within the allotted 6 s) and the time to successful trial completion. We found that all four combinations of strategy and effector resulted in significant control performance compared with chance (Fig. 7*A*). Interestingly, the ARH condition performed significantly better than the other three (IRH, ALH, ILH). This was true when using both trial success rate as a measure of performance (Fig. 7*A*; χ^2^(3, *N* = 1280) = 19.06, *p* = 2.66e-4, Kruskal–Wallis test) and the time to trial completion of the successful trials (Fig. 7*B*; χ^2^(3, *N* = 994) = 16.43, *p* = 9.24e-4, Kruskal–Wallis test).

Hypothetically, there are several possible explanations for why ARH performed better than the other conditions. First, the representation of ARH during training might be more similar to its corresponding representation during online control, either at a single-unit level or a population level. This would lead to the decoder trained on the ARH training data performing better during online control. Second, more units could be tuned to ARH, resulting in a larger signal and thus better control performance. Finally, the ARH-tuned units might be more reliably tuned than the other conditions, having more consistent neural responses trial to trial on a unit-by-unit basis, also leading to a larger signal.

In regard to the first possibility, our above results already show that there are no differences in how well the different movement representations are maintained. Not only are each of the representations equally well maintained, but their structure is maintained too (Figs. 2*B–E*, 4).

Thus, we first sought to explain the performance differences simply as a function of the number of units used for each decoder. There were significant differences in how many of the units were tuned to each of the four conditions. Focusing on the training data, a test of equal tuning percentages for all four conditions only trended toward significance (Fig. 2*A*; χ^2^(3, *N* = 2372) = 5.86, *p* = 0.12, Kruskal–Wallis test on the significance values of the linear model fits). However, a test of ARH compared with all other conditions showed a significant difference (χ^2^(1, *N* = 2372) = 4.35, *p* = 0.037, Kruskal–Wallis test), consistent with the observed performance differences.

We next asked whether there were any differences in reliability of tuning of the units tuned to each condition. The performance differences could be driven not only by the greater number of ARH-tuned units, but also the ARH tuned units being more reliably tuned. We used the *R*^2^ value of the linear model fits computed previously as a measure of the reliability of tuning. Once again focusing on only the training data, while ARH tended to have more tuned units, on average, ARH-tuned units were not any more reliably tuned than units tuned to other conditions (Fig. 7*C*; χ^2^(3, *N* = 333) = 1.99, *p* = 0.58, Kruskal–Wallis test). These results suggest that on a unit-by-unit basis, there is nothing qualitatively special about the units tuned to ARH compared with the units tuned to other conditions.

Previously, we found that the correlations of the movement conditions between training and online control were also all comparable (Fig. 2*F*) and that the structure of the representations is largely maintained between training and online control (Figs. 4, 5). In light of these results, it makes sense that any performance differences between the conditions existing during training might carry over to online control. In other words, it should be possible to predict the online control performance trends based on solely looking at the training data. To test this, we examined the cross-validated *R*^2^ value of the decoder of each movement condition (decoders trained on data from the training runs, *R*^2^ computed from regression of the predicted to the ideal trajectories; Fig. 7*D*). ARH had a significantly higher cross-validated *R*^2^ value than the other conditions (χ^2^(3, *N* = 32) = 10.69, *p* = 0.014, Kruskal–Wallis test). This is the same trend as found in our performance measures (Fig. 7*A*,*B*). Consistent with the structure maintained result, the properties of the movement conditions during training were also preserved in online control.

## Discussion

In this study, we recorded from human AIP of a tetraplegic participant and investigated how well the structure of the mixed representations was maintained between different task contexts. Focusing on the task contexts of open-loop motor imagery (training) and closed-loop cortical control (online), we found that the different tested effectors (left and right hand) and cognitive strategies (imagine and attempt) could all be used for control, with performance differences primarily due to differences in the number of units tuned to each movement condition during training.

### Consistency of representations across different task contexts

Our laboratory recently found partially mixed representations in human AIP, with strategy and body side variables mixed and functionally segregated by body part (Zhang et al., 2017). Consistent with that study, we found that a significant fraction of our recorded neural population encoded the tested movement conditions during training (Fig. 2*A*). Furthermore, populations tuned to each of the variables were overlapping, with units having varying degrees of specificity to one variable over another (Fig. 2*B*–*E*), also consistent with the larger overlap between the variables found in the study by Zhang et al. (2017).

Extending our previous study, we examined these representations not just in the absence of closed-loop BMI control as the previous study did (training), but also during online control, investigating the degree to which the representations change from one to the other. We found that the representations of the movement conditions were largely maintained between training and online control. Individual units tended to keep their tuning and specificity between training and online control (Fig. 6). Population representations as a whole also stayed relatively similar between the two contexts (Fig. 2*F*). Furthermore, we found that not only were the representations of the tested movement conditions largely maintained, but the structure of their representations (i.e., the relationship between the representations) was maintained and the movement conditions could be differentiated during both training and online control (Figs. 4*A*,*B*, 5*A*,*B*). This is especially remarkable because of the movement conditions tested. The cognitive strategy (imagine vs attempt) and body side (left vs right) variables have significantly more overlapping neural representations than body part (hand vs shoulder; Zhang et al., 2017). Thus, the movement conditions studied here are more difficult to differentiate. The structure is also maintained despite the differences between the training and online control tasks (two targets vs three targets; Fig. 1), making the result even more significant.

The relative maintenance of representations from training to online control is consistent with the ability to use recordings from AIP for brain control (Aflalo et al., 2015). While there can certainly be tuning changes in some of the units when moving to online control (Taylor et al., 2002; Chase et al., 2009; Cunningham et al., 2011), a percentage of the population must be relatively consistent in its behavior for a decoder to generalize and perform online. That said, this previous consistency was found in the directional tuning preferences of the neural population. It is not obvious a priori that the representations of all the different movement conditions would be preserved between training and online control. For example, there is good reason for the brain to largely preserve neural representations of movement direction across open-loop and closed-loop conditions. The distinction between other aspects of movement (e.g., cognitive strategies of imagine or attempt) when the subject is actively controlling the cursor is not directly task relevant and the brain has less incentive to maintain the distinction. Studies in NHP PFC and PPC have observed context-dependent tuning changes before. Specifically, they found that some variables can become more/less strongly tuned in certain contexts than others, while other variables remain similarly well represented regardless of context (Wallis et al., 2001; Gail et al., 2009; Siegel et al., 2015).

Our results in Figures 4, *A* and *B*, and 5, *A* and *B*, demonstrate condition-dependent intention signals in AIP, with both the body side and cognitive strategy variables remaining distinct during online control. Representations were not only still distinct during online control, but also in such a way that the relationships between the representations are consistent between the two contexts. Our finding that there is strong condition specificity, under two very different behavioral contexts, indicates that in humans, as in NHPs, the specific movement intention is a guiding feature of the population structure in PPC.

The maintenance of the distinctions between the movement conditions and structure of their representations also suggests that it is possible to control multiple effectors, at least when the movements are performed individually, while recording from a single brain area. We were able to successfully decode not just the onset of the movements (i.e., squeeze and release), but also the body side and cognitive strategy used in the movement. To the best of our knowledge, past studies have only looked at BMI control using multiple effectors (e.g., bimanual control) in the context of multiple brain areas (Ifft et al., 2013). Note, however, that generalization between training and online control is not perfect (Figs. 4, 5) as classification accuracy distinguishing the cognitive strategy and the body side drops slightly. This indicates that classification parameters should be updated to optimize classification accuracy during online control.

### Performance of different effectors during online control

In this study, we also assessed the online control performance of four movement conditions (attempted/imagined movements of the left/right hand) and found that all performed significantly above chance (Fig. 7*A*,*B*). While the representations of each of the conditions were similarly well maintained between training and online control (Fig. 2*F*), attempted movements of the right hand performed significantly better than the other movement conditions. The reliability of individual units also did not significantly differ based on the movement condition (Fig. 7*C*).

The primary difference in the representations of ARH movement compared with the others was the greater proportion of tuned units (Fig. 2*A*) found in the training data. The maintenance of the structure of the representations allows these differences to carry over to online control. Since the relationship between the representations does not change, the pattern of performance differences predicted during training would not significantly change during online control either. In other words, the maintenance of the structure makes it possible to predict relative online control performance based on offline training data, as demonstrated by the similarity in the trends between the decoder cross-validated *R*^2^ value (Fig. 7*D*) and the online control performance (Fig. 7*A*,*B*).

The greater proportion of units tuned to ARH movement is also consistent with our array recording location. The array is located in left AIP, a region traditionally thought to encode grasp information of the contralateral limb more specifically (Murata et al., 2000; Chang et al., 2008). Thus, a preference for the right hand would be plausible in this brain region.

Furthermore, some studies on BMI control using EEG and other recording technologies found attempted movements to perform better than imagined movements (López-Larraz et al., 2012; Blokland et al., 2014). This is consistent with our finding that the attempt strategy performed better than the imagine strategy in the right hand (Fig. 7*A*,*B*) as well as the bias toward attempted over imagined right-hand movements in the degree of specificity of the individual units during online control (Fig. 2*C*).

This study is part of a clinical trial composed of a variety of experimental tasks involving BMI control beyond those presented here. Most of those studies involved attempted movements of the right hand. Thus, at the time of data collection NS was significantly more practiced using attempted right-hand movements for control than imagined right-hand movements or movements of the left hand. Some studies have shown that neurons can change their tuning behavior and even reorganize with extensive practice (Matsuzaka et al., 2007; Ganguly and Carmena, 2010). The greater amount of practice with the right hand might also have affected our control performance results and would be an interesting subject for future study. However, performance differences between effectors and strategies was relatively small, suggesting that extensive practice has, at most, a marginal effect on brain control performance.

Our results suggest that the structure of the mixed representations is largely maintained across changes in task context. While our study focused primarily on the BMI contexts of training and online control, our results also have implications for other changes in task context as well. Parts of parietal cortex have been known to modulate their neural responses as a function of other contextual task variables, such as the reward or value of an action (Platt and Glimcher, 1999; Iyer et al., 2010), or the modality of the stimuli cueing an action (Raposo et al., 2014). The generalization of the structure of the mixed representations between training and online control suggests that the structure might generalize between these other task contexts as well and thus be a robust organizing feature of neural coding.

## Synthesis

Reviewing Editor: Trevor Drew, University of Montreal Decisions are customarily a result of the Reviewing Editor and the peer reviewers coming together and discussing their recommendations until a consensus is reached. When revisions are invited, a fact-based synthesis statement explaining their decision and outlining what is needed to prepare a revision will be listed below. Note: If this manuscript was transferred from JNeurosci and a decision was made to accept the manuscript without peer review, a brief statement to this effect will instead be what is listed below.

The authors carefully addressed concerns of the previous review. The reviewer has no further comments or questions.
